# Estimated projection of incidence and mortality of alcohol-related liver disease in China from 2022 to 2040: a modeling study

**DOI:** 10.1186/s12916-023-02984-7

**Published:** 2023-07-27

**Authors:** Meiyu Wu, Shuxia Qin, Chongqing Tan, Sini Li, Ouyang Xie, Xiaomin Wan

**Affiliations:** 1 Department of Pharmacy, The Second Xiangya Hospital, Central South University, Changsha, 410011 Hunan China; 2 Institute of Clinical Pharmacy, Central South University, Changsha, 410011 Hunan China; 3 The Nethersole School of Nursing, Faculty of Medicine, The Chinese University of Hong Kong, Hong Kong Special Administrative Region, China

**Keywords:** Alcohol-related liver disease, Burden, China

## Abstract

**Background:**

China has one of the highest numbers of liver disease cases in the world, including 6.4 million cirrhosis associated with alcohol-related liver disease (ALD) cases. However, there is still a lack of urgent awareness about the growth of alcohol consumption and the increased burden of ALD in China. Therefore, we aimed to project the potential impact of changes in alcohol consumption on the burden of ALD in China up to 2040 under different scenarios.

**Methods:**

We developed a Markov model to simulate the natural history of ALD until 2040 in China. We estimated the incidence and mortality of alcohol-related cirrhosis and hepatocellular carcinoma between 2022 and 2040 under four projected scenarios: status quo scenario and scenarios with a 2%, 4%, and 8% annual decrease in excessive alcohol consumption, respectively.

**Results:**

Under the status quo scenario, the cumulative new cases of cirrhosis from 2022 to 2040 was projected to be 3.61 million (95% UI 3.03–4.44 million), resulting in a cumulative 1.96 million (1.66–2.32 million) deaths from alcohol-related cirrhosis and hepatocellular carcinoma. However, a 2% annual reduction in excessive alcohol consumption was expected to avert 0.3 million deaths associated with ALD, and a 4% annual reduction was projected to prevent about 1.36 million new cases of cirrhosis and prevent 0.5 million ALD-related deaths. Moreover, an 8% annual reduction would prevent about 2 million new cases of cirrhosis and 0.82 million deaths.

**Conclusions:**

Without any substantial change in alcohol attitudes and policies to regulate excessive drinking, the disease burden of ALD in China will increase enormously. Strengthening the implementation of alcohol restriction interventions is critical and urgent to reduce the impact of ALD on the Chinese population.

**Supplementary Information:**

The online version contains supplementary material available at 10.1186/s12916-023-02984-7.

## Background

Alcohol-related liver disease (ALD) refers to a range of liver manifestations, including fatty liver disease, alcoholic hepatitis, liver fibrosis, and cirrhosis with its complications [1–4]. Alcohol-related cirrhosis may further progress to hepatocellular carcinoma [5]. Excessive consumption of alcohol is the primary cause of ALD and its associated deaths [6]. Patients with advanced ALD have a poor prognosis, with a median survival time of only 1–2 years [7]. In 2017, alcohol-related cirrhosis affected 26.1 million people and caused 332,268 deaths worldwide, with prevalence and mortality rates of 318.1 and 4.1 per 100,000 population, respectively [8]. The prevalence of alcohol-related cirrhosis in China was estimated at 328.4 per 100,000 population, which was lower than that in Russia (709.7 per 100,000 population) but higher than that in Japan (313.1 per 100,000 population) and Singapore (267 per 100,000 population). However, due to China’s large total population, the number of alcohol-related cirrhosis cases was as high as 6.4 million [8]. According to the World Health Organization (WHO), the alcohol per capita consumption (APC) (liters of pure alcohol, 15 + years of age) in Europe declined from 12.3 l in 2005 to 9.8 l in 2016. In contrast, China experienced rapidly growing APC, which rose from 4.1 to 7.2 l in the same period, a 75% increase [9]. Although alcohol consumption in China is lower than in many high- and middle-income countries, there is a growing concern about its rapid increase due to the country’s economic growth and lifestyle changes. This alarming trend has the potential to further exacerbate the disease burden of ALD [10]. Despite this alarming trend, healthcare providers and the general population in China lack awareness about the growth of alcohol consumption and its association with ALD [11]. Policymakers in China appear to prioritize tobacco control over alcohol restriction. For example, the Healthy China 2030 strategy aims to reduce the smoking rate of people over 15 years old to 20% by 2030 and identifies tobacco control as a priority, but it lacks specific goals and measures for alcohol restriction [12, 13].

There are several studies on the impact of reduced alcohol consumption on liver outcomes [14–16]. However, there is a lack of evidence on the population-level impact of reducing excessive alcohol consumption on the disease burden of ALD in China, where there is a large drinking population and alcohol consumption is rapidly increasing. Projections of ALD and mortality based on various alcohol restriction scenarios can quantify the effectiveness of alcohol restriction policies and aid in setting goals for such policies. Moreover, these projections can provide valuable insights into the effects of changes in drinking behavior in the country. Therefore, the purpose of this study is to project the trends of alcohol-related cirrhosis and mortality in China up to 2040 under four different potential alcohol consumption levels, which provide policymakers with evidence-based guidance for implementing effective alcohol restriction policies.

## Methods

### Overview

We developed a Markov model to simulate the natural history (i.e., the progression of a disease process in an individual over time without treatment or alcohol policy intervention [17]) of ALD caused by excessive drinking in China based on previously published studies [1, 16, 18]. In this modeling study, the simulated population consists of people living in China between 2000 and 2040, involving males and females born during 1915–2000. We calibrated the probability of drinking for each age group to fit the age- and sex-specific prevalence of excessive alcohol consumption from China National Nutrition and Health Survey (CNNHS) for 2002 and 2012 [19, 20]. To validate our natural history model, we compared the results predicted by the model with the annual mortality of liver cancer due to alcohol use and annual mortality of cirrhosis and other chronic liver diseases due to alcohol use in China, as reported by the Global Burden of Disease Project (GBD) for the years 2010–2019, respectively [21]. We then used the natural history model to explore the impact of different levels of alcohol restrictions on the temporal trends of alcohol-related cirrhosis and hepatocellular carcinoma from 2022 to 2040. The model was developed using R software (R version 4.0.5; http://www.r-project.org), with a yearly time-step.

### Natural history of alcohol-related liver disease

**Fig. 1:**
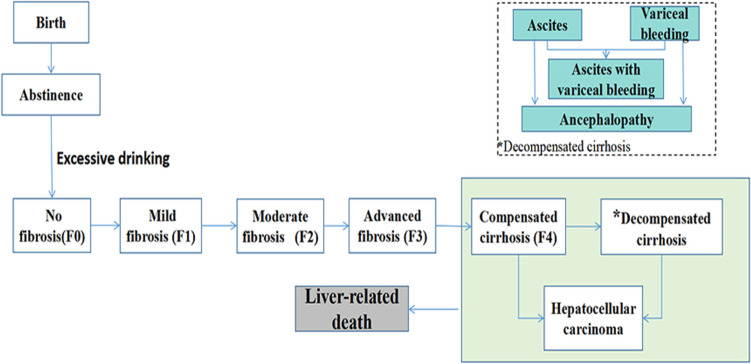
Model structure of alcohol-related liver disease progression. The model tracked the virtual populations until death, which could occur in all health states at any time

In this model, each cohort was followed from birth to death, and the progression of ALD was simulated. At any given time, the virtual patients could remain in their current state or transition to another state on the basis of transition probabilities. We postulated that it is only people aged 18 years and over who are likely to drink excessively and to move from abstinence to excessive drinking. Excessive drinkers could progress from no fibrosis (F0) to varying degrees of fibrosis and cirrhosis (F1: mild fibrosis; F2: moderate fibrosis; F3: advanced fibrosis; F4: compensated cirrhosis) (Fig. 1). Subsequently, patients could develop different types of complications (ascites, variceal bleeding, ascites with variceal bleeding, and encephalopathy) and may be diagnosed as decompensated cirrhosis. Hepatocellular carcinoma could develop from compensated cirrhosis or decompensated cirrhosis. Patients could die from background death at any time (death from causes other than liver disease). Furthermore, patients at an advanced stage of the disease would experience an additional death risk due to liver-related mortality, and the risk increases as the liver disease progressed.

### Data collection

The parameters used in our model were mainly obtained from published literature and online databases. We obtained demographic data, such as age and gender distribution, from the National Bureau of Statistics of China [22] (Table S1 in Additional file 1). The demographic changes between 2022 and 2040 were obtained from the medium scenario of the United Nations World Population Prospects 2022, which assumes medium fertility, medium mortality, and medium international migration [23]. Data on the progression and mortality of ALD were obtained from the published literature [24–27]. We extracted the age- and sex-specific background mortality from the China Statistical Yearbook 2021 [22]. We assumed the background mortality is consistent across all projection scenarios and unchanged during the forecasting period (Table S2 in Additional file 1).

To capture the changing prevalence of excessive drinking and distribution of drinkers by age and gender, we calibrated the probability of drinking using published data based on CNNHS [19, 20]. CNNHS is a nationally representative major program that is carried out regularly in China. The CNNHS-2002 surveyed 53,073 Chinese adults in 2002, and the CNNHS-2012 collected 135,824 Chinese adults from 2010 to 2013. These two surveys’ data enable us to estimate the distribution of excessive drinking (defined as the average daily alcohol consumption of more than 25 g for men and more than 15 g for women [28]) by age and gender (Table S1 in Additional file 1).

### Projection scenarios

We constructed four projection scenarios based on potential changes in excessive drinking rates.

The status quo scenario assumed that interventions remained unchanged during the projection period. In this scenario, we projected that the age- and sex-specific excessive drinking rates would continue to increase at the same trend as reported by CNNHS from 2002 to 2012.

The conservative scenario assumed a 2% decrease in the excessive drinking rate per year from 2022 to 2040 in the model. This scenario was based on The Healthy China 2030 strategy, which set a target of reducing the smoking rate from 27.7% in 2015 to 20% by 2030 for China’s policymakers [12, 13].

The strong scenario assumed a 4% decrease in the excessive drinking rate per year from 2022 to 2040. This assumption was based on the success of strict alcohol policies in Russia, which led to a 43% drop in the average per capita alcohol consumption in 14 years, according to the WHO. It is hoped that this success achieved in Russia will inspire other countries to implement effective alcohol policies [9].

The ambitious scenario which builds upon the strong scenario assumed that greater benefits could be achieved with stronger interventions and that more restrictive alcohol policies would decrease the excessive drinking rate by 8% per year from 2022.

### Model outcomes and uncertainty analysis

We used the model to project the incidence and deaths of alcohol-related cirrhosis and hepatocellular carcinoma for 2022–2040 under four different alcohol restriction scenarios. All age-standardized rates were calculated using Segi world standard population [29]. We also estimated the years of life lost (YLLs) due to ALD, which was calculated by multiplying the number of deaths per age by the remaining life expectancy at that age and summing across the year [30]. We chose the life expectancy (i.e., an estimate of the average lifespan that a person of a given age can expect to live) at 80.5 years for women and 74.7 years for man [31].

We performed a probabilistic sensitivity analysis (PSA) by sampling values from the probability distribution of input parameters to capture the joint characterize of the combined uncertainty of model parameters, in particular the prevalence of excessive drinking and transition probabilities. We then calculated the 95% uncertainty intervals (UIs) of the model results for each projection scenario based on 1000 model outputs from PSA.

## Results

### Validation

We validated this model externally by comparing the model results with the deaths of liver cancer due to alcohol use and deaths of cirrhosis and other chronic liver diseases due to alcohol use reported by GBD from 2010 to 2019, respectively. The results of these validations show that the predicted values of the model are consistent with the published data, as presented in Additional file 1 Fig S1.

### Incidence

**Fig. 2:**
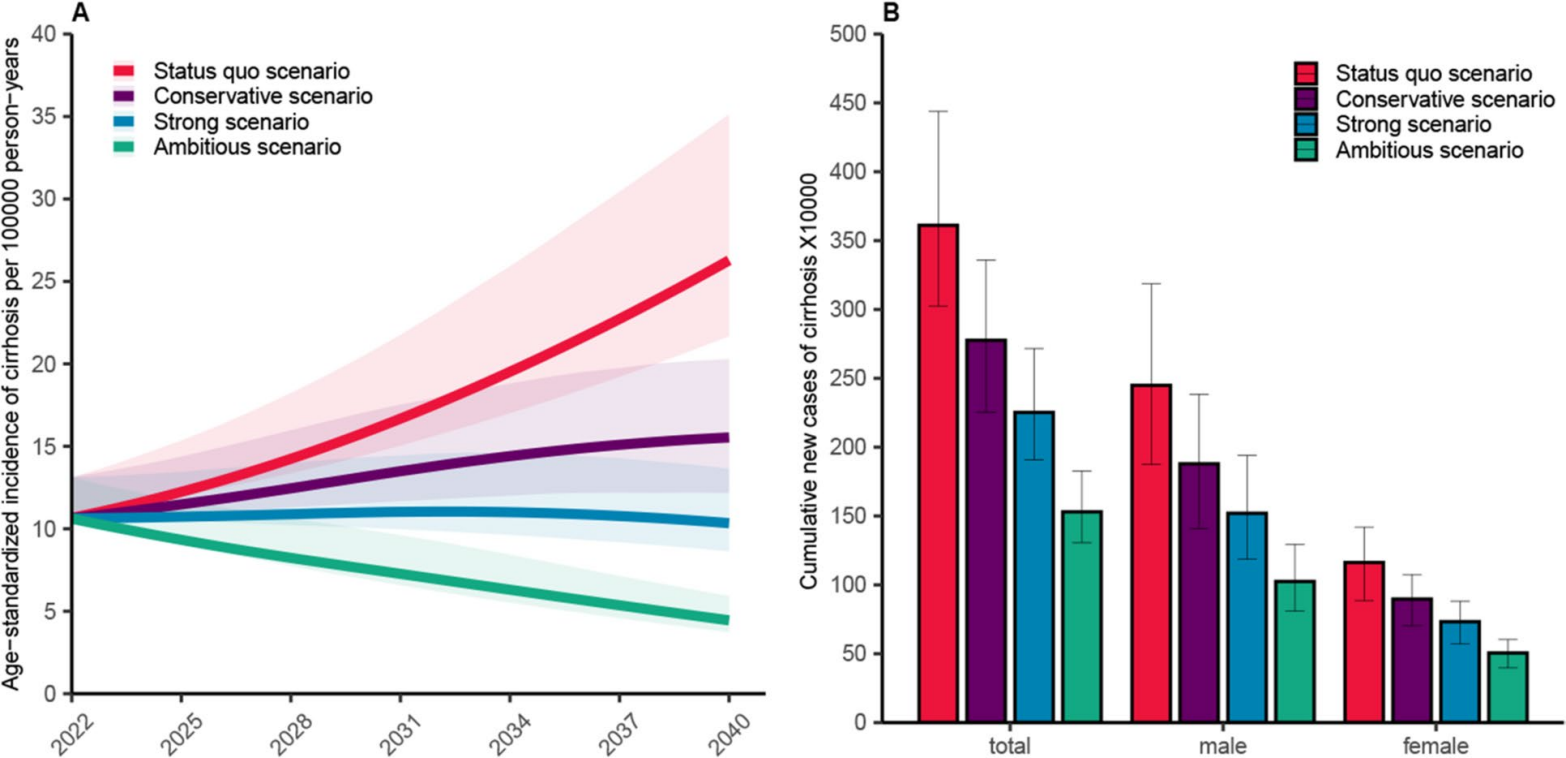
Model projected incidence and cumulative number of alcohol-related cirrhosis in China during 2022–2040. **A** Age-standard incidence rate of cirrhosis, shaded area represents 95% uncertainty interval. **B** The cumulative number of new cases of liver cirrhosis, error bars represent 95% uncertainty interval

Under the status quo scenario (current excessive drinking rates), the age-standardized incidence of alcohol-related cirrhosis is projected to increase from 10.66 (95% UI 10.45–13.12) per 100,000 person-years in 2022 to 26.27 (95% UI 21.67–35.14) per 100,000 person-years in 2040, representing an increase by 146.50% over 2022 [men: from 12.67 (95% UI 12.22–15.90) to 34.95 (95% UI 25.37–49.12) per 100,000 person-years; women: from 8.37 (95% UI 8.33–10.92) per 100,000 person-years to 16.38 (95% UI 12.52–22.67) per 100,000 person-years]. The cumulative incidence of alcohol-related cirrhosis from 2022 to 2040 is projected to be 3,610,931 (95% UI 3,025,219–4,437,429) cases [men: 2,448,603 (95% UI 1,875,188–3,185,085) cases; women: 1,162,328 (95% UI 886,373–1,419,741) cases]. The trend of alcohol-related cirrhosis incidence over time under different scenarios is shown in Fig. 2.

Under the conservative scenario (the excessive drinking rate decreased by 2% per year), the age-standardized incidence of cirrhosis is projected to increase to 15.53 (95% UI 12.18–20.29) per 100,000 population in 2040, a decrease of 40.89% compared with the status quo scenario. A total of 2,775,662 (95% UI 2,254,728–3,359,430) individuals are projected to develop alcohol-related cirrhosis between 2022 and 2040 in the conservative scenario, and 835,269 cases of alcohol-related cirrhosis could be avoided (a decrease of 23.13%) compared with the status quo scenario.

Under the strong scenario (the excessive drinking rate decreased by 4% per year), the age-standardized incidence of cirrhosis is projected to peak at 11.05 (95% UI 9.87–14.57) per 100,000 person-years in 2032, and decreased to 10.34 (95% UI 8.64–13.64) per 100,000 person-years in 2040, resulting in a decrease of 60.64% compared to the status quo scenario. A total of 2,251,880 (95% UI 1,908,370–2,715,670) individuals are projected to develop alcohol-related cirrhosis between 2022 and 2040 under the strong scenario, and 1,359,051 cases of cirrhosis could be prevented, representing a decrease of 37.64% compared to status quo scenario.

Under the ambitious scenario (excessive drinking rate decreased by 8% per year), the age-standardized incidence of alcohol-related cirrhosis is expected to decrease continuously, falling to 4.45 (95% UI 3.72–5.93) per 100,000 person-years by 2040, which represents a 58.2% reduction compared to 2022 levels. The cumulative new cases of alcohol-related cirrhosis are projected to be 1,529,743 (95% UI 1,306,183–1,827,531) under the ambitious scenario, which is 722,137 less than strong scenario (a decrease of 32.07%) and 2,081,188 less than status quo scenario (a decrease of 57.64%).

**Fig. 3:**
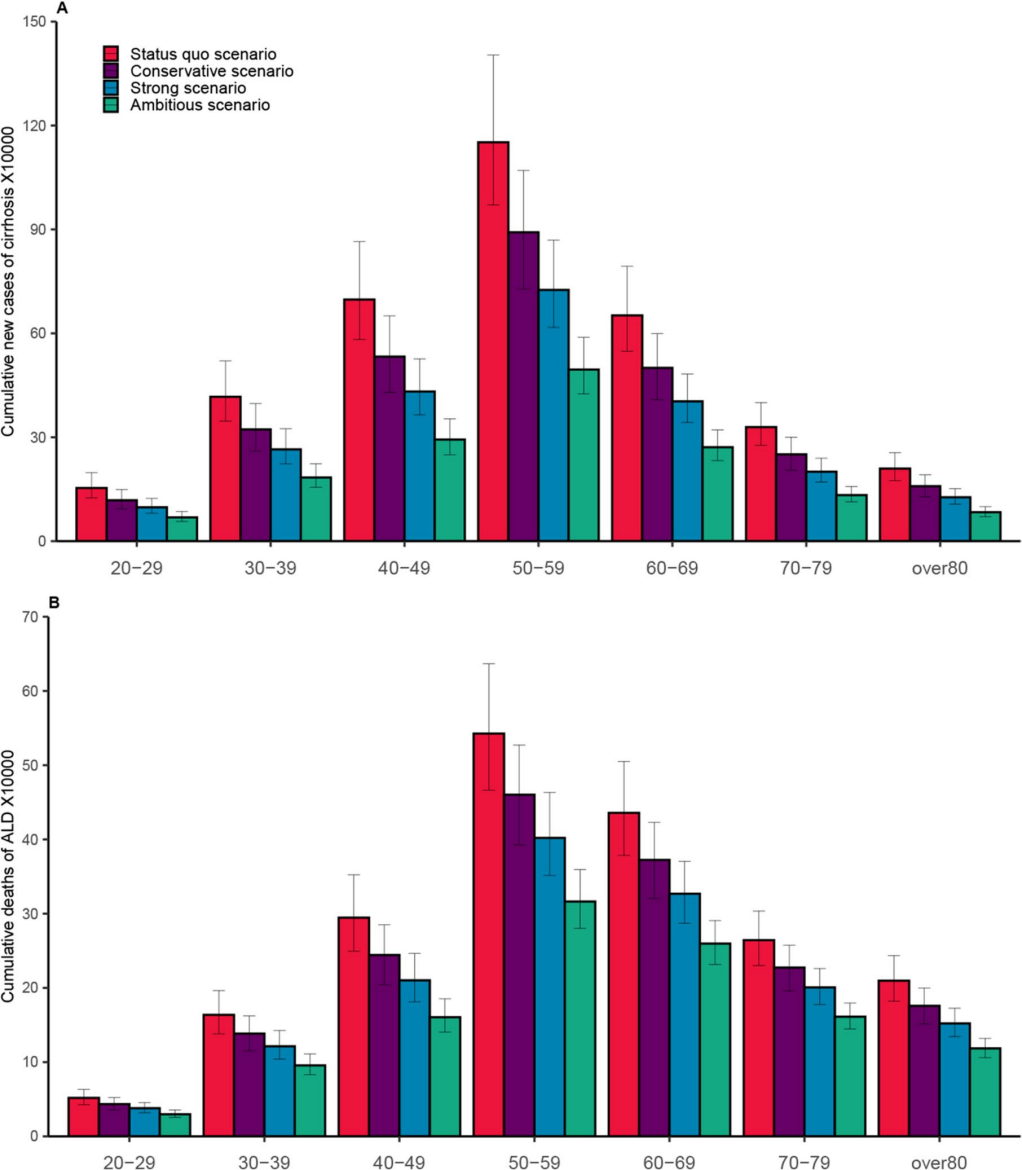
Cumulative incidence of cirrhosis and cumulative deaths of alcohol-related liver disease by age group during 2022–2040. **A** Cumulative incidence of cirrhosis by age group. **B** Cumulative deaths of alcohol-related liver disease by age group. The error bars represent 95% uncertainty interval

In all scenarios, alcohol-related cirrhosis is primarily observed in the age group 40–69 years (69.27%), especially in the age group 50–59 years (32%). The cumulative incidence of alcohol-related cirrhosis between the age of 40 years and 69 years is projected to decrease from 2,501,183 (95% UI 2,101,244–3,062,174) cases in the status quo scenario to 1,561,296 (95% UI 1,325,108–1,878,289 cases in strong scenario (a decrease of 37.58%), and to 1,060,185 (95% UI 907,273–1,262,864 cases in ambitious scenario (a decrease of 57.62%) (Fig. 3A).

### Mortality

**Fig. 4:**
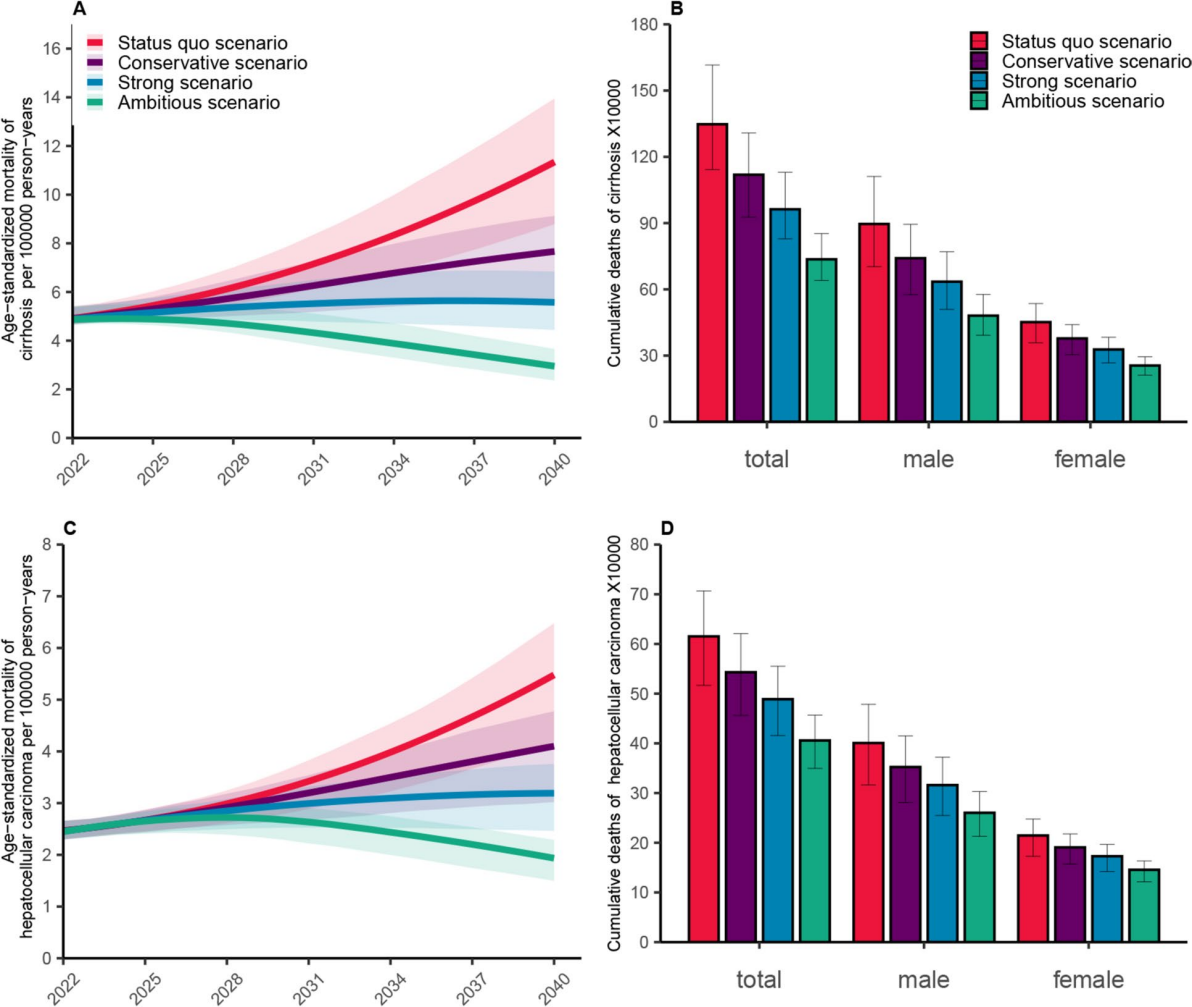
Model projected mortality and cumulative deaths of alcohol-related liver disease in China during 2022–2040. **A** Age-standardized mortality in cirrhosis. **B** The cumulative number of deaths from cirrhosis. **C** Age-standardized mortality in hepatocellular carcinoma. **D** The cumulative number of deaths from hepatocellular carcinoma. The shaded area represents 95% uncertainty interval and the error bars represent 95% uncertainty interval

ALD-related deaths projected by this model included alcohol-related cirrhosis and hepatocellular carcinoma. Under the status quo scenario, the age-standardized deaths of alcohol-related cirrhosis increased from 4.92 (95% UI 4.67–5.41) per 100,000 person-years in 2022 to 11.34 (95% UI 8.79–13.96) per 100,000 person-years in 2040 (an increase of 130.52%), and the age-standardized deaths of hepatocellular carcinoma increased from 2.47 (95% UI 2.30–2.66) per 100,000 person-years in 2022 to 5.48 (95% UI 4.19–6.48) per 100,000 person-years in 2040 (an increase of 122.04%). The cumulative number of ALD-related deaths is projected to be 1,962,795 (95% UI 1,658,405–2,322,781) from 2020 to 2040, with 1,347,602 (95% UI 1,141,751–1,616,023) deaths from cirrhosis and 615,192 (95% UI 516,654–706,759) deaths from hepatocellular carcinoma. Deaths among men are projected to be 1,296,694 (i.e., 66.06% of the total deaths), about 1.94 times that among women (Fig. 4).

Under the conservative scenario, the alcohol-related cirrhosis mortality rate is projected to increase to 7.66 (95% UI 5.69–9.13) per 100,000 and the hepatocellular carcinoma mortality rate to 4.10 (95% UI 3.02–4.78) per 100,000 by 2040. Cumulatively, 1,662,100 (95% UI 1,383,230–1,928,573) people would die from alcohol-related liver disease between 2022 and 2040, 300,695 fewer than in the status quo scenario (a 15.32% reduction).

In the strong scenario, the age-standardized deaths of cirrhosis and hepatocellular carcinoma in 2040 are projected to be 5.57 (95% UI 4.44–6.84) per 100,000 person-years and 3.19 (95% UI 2.46–3.76) per 100,000 person-years, respectively. These rates are 50.88% and 41.78% lower than those in the status quo scenario. Compared to the status quo scenario, reducing the excessive drinking rate by 4% annually between 2022 and 2040 would prevent 385,041 deaths due to cirrhosis and 126,359 deaths due to hepatocellular carcinoma resulting from excessive alcohol consumption. Of these prevented deaths, 67.66% are projected to be men.

Under the ambitious scenario, it is projected that 1,141,830 (95% UI 990,292–1,309,442) individuals would die from ALD between 2022 and 2040, of which 736,093 (94% UI 640,666–852,429) would die from cirrhosis and 405,737 (95% UI 349,626–457,013) would die from hepatocellular carcinoma. These figures represent 309,565 fewer deaths than those projected under the strong scenario (a decrease of 21.33%) and 820,965 fewer deaths than the status quo scenario (a decrease of 41.83%).

In all scenarios, the 50–69 age group is projected to have the highest proportion of deaths, accounting for 49.85% of all cases. However, the cumulative number of ALD-related deaths in this age group is expected to decline from 978,481 (95% UI 845,086–1,141,776) in the status quo scenario to 729,000 (95% UI 638,658–833,766) in the strong scenario (a decrease of 25.50%) and to 576,084 (95% UI 511,892–649,985) in the ambitious scenario (a decrease of 41.12%) (Fig. 3B).

### YLLs

**Fig. 5:**
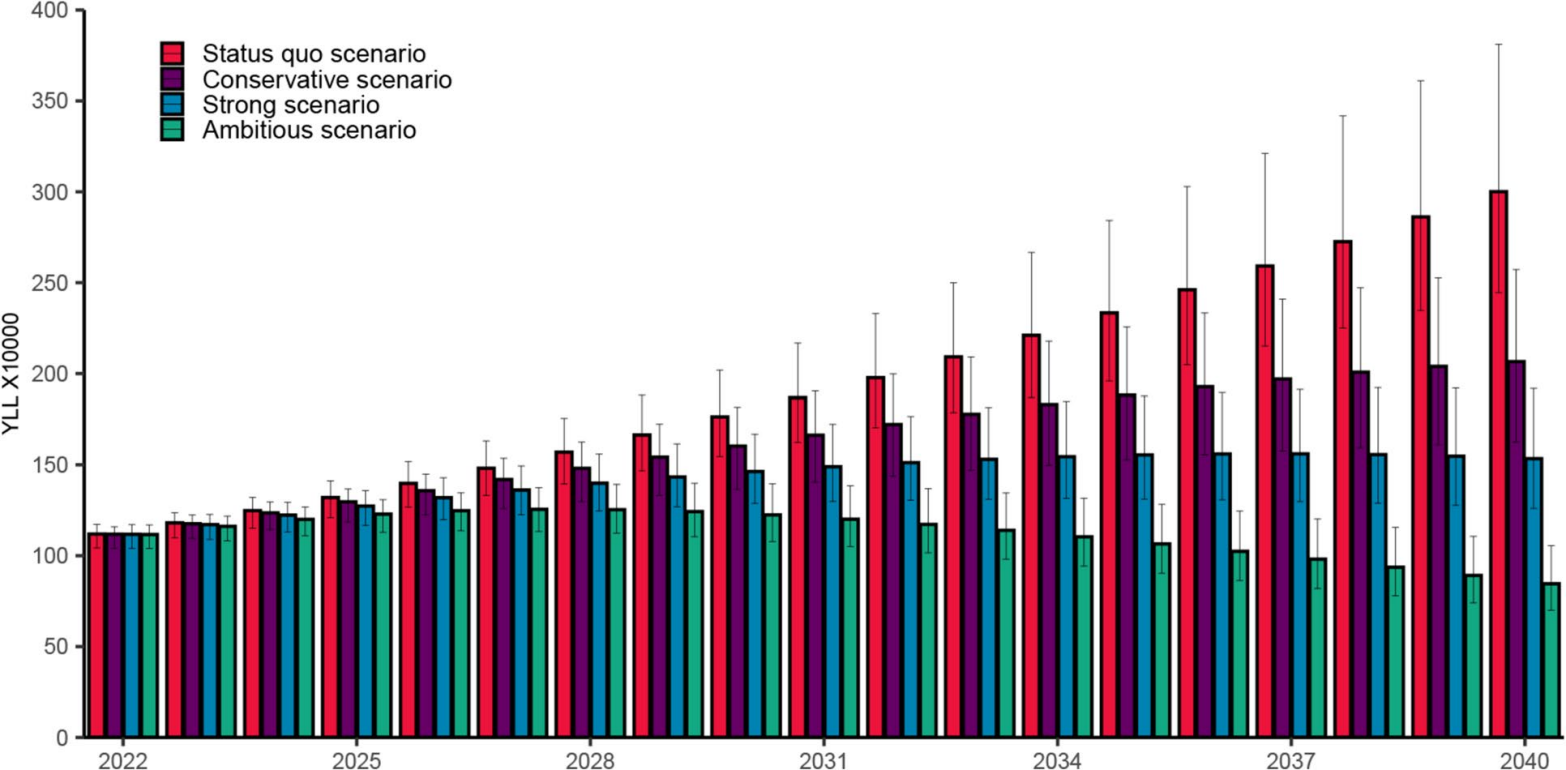
Model projected YLL due to alcohol-related liver disease under different projection scenarios in China during 2022–2040. The error bars represent 95% uncertainty interval. YLL, years of life lost

It is estimated that 1,118,927 (95% UI 1,042,213–1,171,701) YLLs would be attributed to ALD in 2022 and would increase to 3,000,472 (95% UI 2,444,927–3,811,120) YLLs in 2040 in the status quo scenario, resulting in a cumulative 36.87 million (95% UI 31.29–43.44) YLLs between 2022 and 2040. The conservative scenario is expected to result in 31.11 million (95% UI 26.24–35.94) YLLs over the next 19 years. The accumulated YLLs in the strong scenario for the same period are projected to be 9.72 million fewer than status quo scenario (a decrease of 26.36%), resulting in 27.15 million (95% UI 23.63–31.41) YLLs attributable to ALD. The ambitious scenario is projected to result in 21.29 million (95% UI 18.73–24.33) YLLs, which is 15.58 million fewer than the status quo scenario (a 42.26% decrease) (Fig. 5).

## Discussion

The negative impact of the rising rate of excessive drinking on public health is increasing in China. Maintaining the current drinking trends, the results of the present study showed that from 2022 to 2040, more than 3.6 million individuals would develop alcoholic cirrhosis, and there would be nearly 2 million ALD-related deaths. However, we found that a 2% annual reduction in excessive drinking rates could avert 0.3 million deaths associated with ALD. Moreover, 1.36 million new cases of alcoholic cirrhosis and 0.5 million ALD-related deaths would be prevented if the rate of excessive drinking decreased by 4% annually. The burden of ALD was further alleviated when the rate of excessive drinking decreased by 8% annually, preventing almost 2 million individuals from developing cirrhosis and 0.8 million patients from ALD-related death. Therefore, there is an urgent need to formulate effective policies to curb the trend of alcohol consumption. While our study aims to present a range of scenarios with different levels of ambition and assumptions to explore the potential outcomes of alcohol policies, it is important to note that the strong scenario is an optimistic one, while the ambitious scenario would be difficult to achieve and may require very restrictive measures.

Compared to men, women are less tolerant of alcohol and fibrosis progresses more rapidly in women with ALD [24, 32]. However, from the results of this study, the morbidity and mortality in men far exceed that in women. This is mainly because men have more social opportunities to drink than women, due to the cultural and social norms that shape the drinking occasions and expectations in China. Drinking is often associated with business, networking, and entertainment activities that are dominated by men. Moreover, drinking is seen as a sign of masculinity and status among men, while women are expected to drink less or abstain from alcohol. These factors result in a higher proportion of men drinking than women in China [10]. It is worth noting that in recent years, with the enhancement of female consciousness, more females devote themselves to work, so the proportion of female drinkers has risen sharply. The substantial increase in the number of alcohol drinkers may significantly increase the proportion of women in the disease burden of ALD in the future. Although men are the main component of excessive drinkers and deserve more attention in alcohol policy, health providers still cannot ignore the impact of potential demographic changes of female drinkers.

China has a significant impact on the global burden of liver disease due to its large liver disease population [33]. Without any change in the trend of alcohol consumption, our model projected that the number of patients with alcohol-related cirrhosis and hepatocellular carcinoma would increase at an alarming rate in the coming decades. A study investigating the burden of liver diseases in China revealed that viral hepatitis constitutes a significant proportion of liver diseases. However, achieving the goal of reducing China’s global prevalence of liver disease will be difficult unless there are improvements in the incidence and progression of ALD and nonalcoholic fatty liver disease (NAFLD) [33]. Therefore, controlling the prevalence of hepatitis C and hepatitis B alone may not be sufficient for China to shed its status as a major contributor to global liver disease. Due to the limited treatment options available, and the fact that only a few drugs have been approved for ALD, abstinence and liver transplantation are currently the most effective means of improving survival in end-stage ALD [34, 35]. Therefore, a combination of clinical interventions and effective alcohol restriction measures could be a suitable approach to reverse the trend of ALD. The results showed a high incidence and mortality of ALD in the 40–69 age group. As the population ages and life expectancy increases, the risk of developing and dying from ALD in later life may also increase in the long term due to a decrease in the risk of competitive death (background death). Therefore, early interventions should prioritize this population.

ALD-related death is a serious consequence of alcohol consumption [9]. To the best of our knowledge, this is the first study on projecting the future disease burden of ALD in China. By projecting its disease burden in the next 19 years, our study emphasizes the need to strengthen the implementation of alcohol restriction policies. However, compared to alcohol, the Chinese public and policymakers appear to be more concerned about the harm caused by cigarettes. In recent years, China has made significant progress in tobacco control by implementing numerous bans and regulations and investing significant resources to reduce smoking rates [36]. The disease burden caused by smoking is expected to decline sharply in the foreseeable future. We believe that similar resources should also be invested in curbing the rising trend of ALD. Moreover, restricting alcohol consumption not only benefits ALD but also helps prevent and control other major noncommunicable diseases (e.g., cardiovascular diseases and cancer) and prevents alcohol-induced injuries (e.g., traffic injuries and homicides) [37, 38].

The results of an economic analysis conducted by WHO showed that implementing the most cost-effective alcohol restriction actions yields a higher return on investment than similar investments in tobacco control [9]. Although additional research is needed to explore the relative importance and economy of alcohol policy in China, policies aimed at increasing alcohol prices, regulating alcohol supply, and restricting alcohol marketing are likely to be of significant importance in effectively reducing alcohol consumption [39, 40]. In China, the low cost of alcohol is one of the factors that contribute to excessive drinking. Raising the price of alcohol through taxation has been effective in many countries and should be explored as a key potential strategy in China [39]. In addition, Yu et al. have suggested that taxing alcohol could have a dual benefit by reducing both alcohol and cigarette consumption [41].

Limiting alcohol supply has been implemented in many countries and has proven to be effective in reducing excessive drinking and related harms [40]. Restricting the availability of alcohol can be a useful strategy for China to control alcohol consumption, including restricting the trading time of alcohol, restricting the issuance of licenses, and establishing the legal minimum age for the purchase and consumption of alcohol. However, the efforts of relevant policies in China are currently lagging behind the innovation of alcohol marketing. Hence, the government needs to strengthen the establishment and implementation of alcohol marketing laws and regulations to control alcohol marketing more effectively. For example, restrictions on alcohol advertising and product placement could be implemented. In addition, community action has proved to be an effective way to reduce alcohol-related problems [42]. Community supervision can prevent the illegal sale of alcohol. Through public education, a sustained understanding and awareness of the harmful effects of excessive drinking can be established among the general population in China.

### Limitation

As with all studies that project future disease burden, our projections are also subject to model assumptions and uncertainty. Our study has several limitations. First, the progression probabilities for ALD in our model were mainly derived from published studies conducted in other countries, which may not accurately reflect the progression risk of excessive drinkers in China. However, we conducted a PSA to evaluate the impact of parameter uncertainty, and the results indicated that the overall incidence and death trends were robust. Second, our model did not consider other risk factors, such as hepatitis B virus and hepatitis C virus, that could promote the progression of liver fibrosis and cirrhosis, potentially leading to an underestimation of the future disease burden. Third, our model did not consider the possibility of varying mortality rates over time or across different scenarios, which could further increase the uncertainty of the results. Fourth, we assumed that the initial age of excessive drinking was 18 years or older, which may have omitted the disease burden in the younger group. However, given that the incidence of alcohol-related cirrhosis and ALD-related deaths mainly occurred in cohorts over 40 years old, the omission of younger excessive drinkers would have minimal impact on our overall results. Fifth, we assumed that alcohol consumption decreased uniformly across age groups in the projection scenario, but different age groups may respond differently to alcohol restriction policies, potentially leading to varying effects on alcohol restriction. Sixth, we used only survey data for the model. There is a discrepancy between the survey-based per capita consumption (2.2 l in 2002 and 4.45 l in 2012) data and the recorded per capita consumption data (3.2 l in 2002 and 5.5 l in 2012) [9]. While we have conducted PSA to account for the uncertainty associated with using only survey data, this discrepancy may still result in a conservative estimate of the burden of ALD. Finally, we assumed only one probability for any drinking above 25 g/15 g per day for men/women. This simplification may introduce increased uncertainty into the results, as the risk of disease progression can vary among different levels of alcohol consumption. Future research should consider incorporating more refined probabilities that account for varying levels of alcohol consumption.

## Conclusions

In conclusion, our mathematical model projects an increase in the incidence and mortality of alcohol-related cirrhosis and hepatocellular carcinoma over the next 19 years. However, reducing alcohol consumption can effectively alleviate the burden of alcohol-related diseases, resulting in improved health outcomes and increased economic productivity.

## Supplementary Information

**Additional file 1.** More details of the model construction and input parameters. **Table S1.** Baseline data source summary for the model. **Table S2.** Key parameters for the model. **Fig S1.** Comparison of model predictions and GBD reported values. **Fig S2.** The number and prevalence of excessive alcohol consumption for all projected scenarios.

## Data Availability

The data generating the findings of this article are included within the article and its additional files.

## Abbreviations

95% UI: 95% Uncertainty interval
ALD: Alcohol-related liver disease
CNNHS: China National Nutrition and Health Survey
GBD: Global burden of disease project
HCC: Hepatocellular carcinoma
PSA: Probabilistic sensitivity analysis
YLLs: Years of life lost

## References

[1] Li YM, Fan JG (2019). Guidelines of prevention and treatment for alcoholic liver disease (2018, China). J Dig Dis.

[2] Horrell J, Callaghan L, Dhanda A (2022). Alcohol misuse in patients with alcohol-related liver disease: how can we do better? A narrative review of the literature. Alcohol Clin Exp Res.

[3] Singal AK, Bataller R, Ahn J, Kamath PS, Shah VH (2018). ACG clinical guideline: alcoholic liver disease. Am J Gastroenterol.

[4] Ha Y, Jeong I, Kim TH (2022). Alcohol-related liver disease: an overview on pathophysiology, diagnosis and therapeutic perspectives. Biomedicines.

[5] Ganne-Carrie N, Nahon P (2019). Hepatocellular carcinoma in the setting of alcohol-related liver disease. J Hepatol.

[6] Wang H, Ma L, Yin Q, Zhang X, Zhang C (2014). Prevalence of alcoholic liver disease and its association with socioeconomic status in north-eastern China. Alcohol Clin Exp Res.

[7] Bruha R, Dvorak K, Petrtyl J (2012). Alcoholic liver disease. World J Hepatol.

[8] GBD 2017 Cirrhosis Collaborators. The global, regional, and national burden of cirrhosis by cause in 195 countries and territories, 1990–2017: a systematic analysis for the Global Burden of Disease Study 2017. Lancet Gastroenterol Hepatol. 2020;5(3):245–66. 10.1016/S2468-1253(19)30349-8 PMC7026710 31981519

[9] World Health Organization (2018). Global status report on Alcoholand health 2018.

[10] Wang WJ, Xiao P, Xu HQ, Niu JQ, Gao YH (2019). Growing burden of alcoholic liver disease in China: a review. World J Gastroenterol.

[11] Jiang H, Room R, Hao W (2015). Alcohol and related health issues in China: action needed. Lancet Glob Health.

[12] The State Council of the People’s Republic of China. “Healthy China 2030” Plan. 2016. http://www.gov.cn/xinwen/2016-10/25/content_5124174.htm. Accessed 25 May 2023.

[13] World Health Organization. WHO and the Chinese National Health Commission call for immediate action on World No Tobacco Day and urge all to “commit to quit”. https://www.who.int/china/news/detail/26-05-2021-who-and-the-chinese-national-health-commission-call-for-immediate-action-on-world-no-tobacco-day-and-urge-all-to-commit-to-quit#:~:text=The%20Healthy%20China%202030%20strategy%20set%20a%20goal,listed%20tobacco%20control%20as%20one%20of%20its%20priorities. Accessed 25 May 2023.

[14] Gredner T, Niedermaier T, Brenner H, Mons U (2021). Impact of reducing alcohol consumption through price-based policies on cancer incidence in Germany 2020–50-a simulation study. Addiction.

[15] Kilian C, Rovira P, Neufeld M, Ferreira-Borges C, Rumgay H, Soerjomataram I (2021). Modelling the impact of increased alcohol taxation on alcohol-attributable cancers in the WHO European Region. Lancet Reg Health Eur.

[16] Julien J, Ayer T, Bethea ED, Tapper EB, Chhatwal J (2020). Projected prevalence and mortality associated with alcohol-related liver disease in the USA, 2019–40: a modelling study. Lancet Public Health.

[17] Seitz HK, Bataller R, Cortez-Pinto H, Gao B, Gual A, Lackner C (2018). Alcoholic liver disease. Nat Rev Dis Primers.

[18] Asphaug L, Thiele M, Krag A, Melberg HO (2020). Cost-effectiveness of noninvasive screening for alcohol-related liver fibrosis. Hepatology.

[19] Xu X, Zhao L, Fang H, Guo Q, Wang X, Yu W (2016). Status of alcohol drinking among population aged 15 and above in China in 2010–2012. Wei Sheng Yan Jiu.

[20] Ma G, Du S, Hao L, Li Y, Hu X, Kong L (2009). The prevalence of heavy drinking among adults in China. Acta Nutrimenta Sin.

[21] Institute for Health Metrics and Evaluation. Global burden of disease project. https://ghdx.healthdata.org/gbd-results-tool. Accessed 12 Apr 2023.

[22] National Bureau of Statistics. China statistical year book. 2021.

[23] United Nations. World Population prospects. 2022. https://population.un.org/wpp/Download/Standard/Interpolated/. Accessed 25 May 2023.

[24] Poynard T, Mathurin P, Lai CL, Guyader D, Poupon R, Tainturier MH (2003). A comparison of fibrosis progression in chronic liver diseases. J Hepatol.

[25] Jepsen P, Ott P, Andersen PK, Sorensen HT, Vilstrup H (2010). Clinical course of alcoholic liver cirrhosis: a Danish population-based cohort study. Hepatology.

[26] Kanwal F, Khaderi S, Singal AG, Marrero JA, Loo N, Asrani SK, et al. Risk factors for HCC in contemporary cohorts of patients with cirrhosis. Hepatology. 2022;77(3):997–1005. 10.1002/hep.32434 PMC9433461 35229329

[27] Wang CY, Li S (2019). Clinical characteristics and prognosis of 2887 patients with hepatocellular carcinoma: a single center 14 years experience from China. Medicine (Baltimore).

[28] Wang SS, Lay S, Yu HN, Shen SR (2016). Dietary Guidelines for Chinese Residents (2016): comments and comparisons. J Zhejiang Univ Sci B.

[29] Bray F, Guilloux A, Sankila R, Parkin DM (2002). Practical implications of imposing a new world standard population. Cancer Causes Control.

[30] Collaborators GDAI (2020). Global burden of 369 diseases and injuries in 204 countries and territories, 1990–2019: a systematic analysis for the Global Burden of Disease Study 2019. Lancet.

[31] World Health Organization. World Health Statistics 2021. https://www.who.int/data/gho/publications/world-health-statistics. Accessed 13 Apr 2022.

[32] Buzzetti E, Parikh PM, Gerussi A, Tsochatzis E (2017). Gender differences in liver disease and the drug-dose gender gap. Pharmacol Res.

[33] Wang FS, Fan JG, Zhang Z, Gao B, Wang HY (2014). The global burden of liver disease: the major impact of China. Hepatology.

[34] Mellinger JL, Scott WG, DeJonckheere M, Fontana RJ, Volk ML, Lok A (2018). Misconceptions, preferences and barriers to alcohol use disorder treatment in alcohol-related cirrhosis. J Subst Abuse Treat.

[35] Farooq MO, Bataller R (2016). Pathogenesis and management of alcoholic liver disease. Dig Dis.

[36] Xue Y (2020). Smoking cessation programmes in China. Lancet.

[37] Beaglehole R, Bonita R, Horton R, Adams C, Alleyne G, Asaria P (2011). Priority actions for the non-communicable disease crisis. Lancet.

[38] Huang C, Yu H, Koplan JP (2014). Can China diminish its burden of non-communicable diseases and injuries by promoting health in its policies, practices, and incentives?. Lancet.

[39] Tang YL, Xiang XJ, Wang XY, Cubells JF, Babor TF, Hao W (2013). Alcohol and alcohol-related harm in China: policy changes needed. Bull World Health Organ.

[40] Bryden A, Roberts B, McKee M, Petticrew M (2012). A systematic review of the influence on alcohol use of community level availability and marketing of alcohol. Health Place.

[41] Yu X, Abler D (2010). Interactions between cigarette and alcohol consumption in rural China. Eur J Health Econ.

[42] Shakeshaft A, Doran C, Petrie D, Breen C, Havard A, Abudeen A (2014). The effectiveness of community action in reducing risky alcohol consumption and harm: a cluster randomised controlled trial. PLoS Med.

