# Supplementary material for: Estimated projection of incidence and mortality of alcohol-related liver disease in China from 2022 to 2040: a modeling study
Source: BMC Med. 2023 Jul 27;21:277. doi: 10.1186/s12916-023-02984-7 (PMC10375628; doi:10.1186/s12916-023-02984-7)
Supplement: Supplementary file 1 — Additional file 1. More details of the model construction and input parameters. Table S1. Baseline data source summary for the model. Table S2. Key parameters for the model. Fig S1. Comparison of model predictions and GBD reported values. Fig S2. The number and prevalence of excessive alcohol consumption for all projected scenarios. [file 12916_2023_2984_MOESM1_ESM.docx]

S1 Appendix. **More details of the model construction and input parameters**

# S 1.1 Model Construction

We used R software (R version 4.0.5) to build a Markov model to track the population of alcohol-related liver disease (ALD) caused by excessive drinking in China from 2000 to 2040. We used nationally representative demographic data from the Bureau of Statistics of China to simulate the population aged 0-85 years in 2000 as the initial population of the model. (i.e., males and females born during 1915-2000)[1]. The demographic structure of the simulated population changes over time between 2000 and 2040 due to births and deaths[2] We divided the population into three groups based on drinking status: never had excessive drinking (abstinence); excessive drinking; and used to excessive drinking (ex-excessive drinking). We assumed that only those aged ≥ 18 years were able to drink excessively and subsequently move from abstinence to excessive drinking in the model. The probability of drinking varied with age and time and was obtained through calibration. More details about the calibration are described below. Relevant demographic data is presented in Table S1

Our model simulated the disease history of ALD as 12 health states based on previously published studies[3-5]. The progression of fibrosis was divided into four stages based on the severity of fibrosis: no fibrosis (F0); mild fibrosis (F1); moderate fibrosis (F2); advanced fibrosis (F3); compensated cirrhosis (F4). With the aggravation of compensated cirrhosis, it could develop into decompensated cirrhosis with four different types of complications (ascites, variceal bleeding, ascites with variceal bleeding, and encephalopathy) and even hepatocellular carcinoma. Death could occur at any stage of ALD and was classified into two types: deaths from other causes (background death), and liver-related death caused by ALD that occurs in the late stages of ALD. Transition probabilities between the states are obtained from published studies and are shown in Table S2.

We queried the International Classification of Diseases 11th Revision (ICD-11) codes DB94.2 (Alcoholic liver fibrosis), DB94.3 (Alcoholic cirrhosis of liver without hepatitis), 2C12.02 (Hepatocellular carcinoma of liver) to define the relevant outcomes in the model.

# S 1.2 Calibration and validation

Calibration is a method to identify the possible parameters of the model and increase the credibility of the results by comparing the model results with the target epidemiological data. We applied a differential evolution algorithm (DEA), an efficient and powerful algorithm for global optimization, to search for unknown parameters related to excessive drinking probabilities of Chinese male and female within the plausible ranges separately. The calibration targets are age- and gender-specific excessive drinking rates from the China National Nutrition and Health Survey (CNNH) for 2002 and 2012[6, 7] (See Table S1). We used the least squares method to assess the goodness of fit (GOF) between the model outputs and the calibration targets, and selected the parameter sets with the highest GOF as the input of our model.

To evaluate model performance, we conducted a validation study by comparing the the number of liver cancer deaths due to alcohol and the deaths number of cirrhosis and other chronic liver diseases due to alcohol use reported by the Global Burden of Disease project (GBD) from 2010 to 2019 for China in 2017 with the outcomes predicted by our model. The result shows a good external validation between the GBD data and the model outcomes, indicating that the model is well-calibrated. (See Fig S1).

# S 1.3 Projection scenarios

In the status quo scenario, the number of excessive drinkers will increase from 113.31 million in 2022 to 169.24 million in 2040, and the prevalence of excessive drinking will increase from 7.93% in 2022 to 12.05% in 2040. In the conservative scenario, there will be 71.76 million people (5.11% excessive drinking prevalence) with excessive alcohol consumption in 2040. The number of excessive drinkers in 2040 will decrease to 46.02 million (3.28% excessive drinking prevalence) under the strong scenario and to 18.39 million (1.31% excessive drinking prevalence) under the ambitious scenario. The number and prevalence of excessive alcohol consumption for all projected scenarios are shown in Fig S2.

| **Table S1. Baseline data source summary for the model** | | | | |
| --- | --- | --- | --- | --- |
| **Variable** | **Mean value** | **Lower** | **Upper** | **Source** |
| Total male population (thousand) | 656720 | 591048 | 722392 | [1] |
| Total female population (thousand) | 619550 | 557595 | 681505 | [1] |
| Population characteristics of excessive drinking in 2002 (%) | | | | |
| male | 8.4 | 7.6 | 9.2 | [7] |
| female | 0.8 | 0.7 | 0.9 | [7] |
| 18-29 years | 3.3 | 3.0 | 3.6 | [7] |
| 30-44 years | 5.1 | 4.6 | 5.6 | [7] |
| 45-59 years | 6.4 | 5.8 | 7.0 | [7] |
| ≥60 years | 4.4 | 4.0 | 4.8 | [7] |
| Population characteristics of excessive drinking in 2012 (%) | | | | |
| male | 19.0 | 17.1 | 20.9 | [6] |
| female | 1.5 | 1.4 | 1.7 | [6] |
| 18-29 years | 6.8 | 6.1 | 7.5 | [6] |
| 30-44 years | 10.6 | 9.5 | 11.7 | [6] |
| 45-59 years | 15.9 | 14.3 | 17.5 | [6] |
| ≥60 years | 11.6 | 10.4 | 12.8 | [6] |
| Abbreviations: PSA: probabilistic sensitivity analysis | | | | |

| **Table S2. Key parameters for the model** | | | | | | | |
| --- | --- | --- | --- | --- | --- | --- | --- |
| **Transition probabilities (annual)** | **Mean value** | **Lower** | **Upper** | **PSA distribution** | **Parameters 1** | **Parameters 2** | **Source** |
| Male fibrosis progression(F0-F4) | 0.032 | 0.012 | 0.055 | Beta | 24.17 | 731.08 | [8] |
| Female fibrosis progression (F0-F4) | 0.051 | 0.026 | 0.095 | Beta | 23.67 | 440.52 | [8] |
| F4 to Ascites | 0.102 | 0.057 | 0.167 | Beta | 22.35 | 196.75 | [9] |
| F4 to Variceal bleeding | 0.051 | 0.024 | 0.092 | Beta | 23.67 | 440.52 | [9] |
| F4 to Encephalopathy | 0.029 | 0.012 | 0.049 | Beta | 24.25 | 811.82 | [9] |
| F4 to HCC | 0.027 | 0.014 | 0.049 | Beta | 24.30 | 875.63 | [10] |
| F4 to Liver-related death | 0.057 | 0.026 | 0.093 | Beta | 23.52 | 389.08 | [9] |
| Ascites to Ascites with variceal bleeding | 0.078 | 0.035 | 0.141 | Beta | 22.97 | 271.54 | [9] |
| Ascites to Encephalopathy | 0.083 | 0.041 | 0.142 | Beta | 22.84 | 252.36 | [9] |
| Ascites to HCC | 0.027 | 0.014 | 0.049 | Beta | 24.30 | 875.63 | [10] |
| Ascites to Liver-related death | 0.071 | 0.031 | 0.122 | Beta | 23.15 | 302.96 | [9] |
| Variceal bleeding to Ascites with variceal bleeding | 0.102 | 0.038 | 0.192 | Beta | 22.35 | 196.75 | [9] |
| Variceal bleeding to Encephalopathy | 0.085 | 0.033 | 0.144 | Beta | 22.79 | 245.33 | [9] |
| Variceal bleeding to HCC | 0.027 | 0.014 | 0.049 | Beta | 24.30 | 875.63 | [10] |
| Variceal bleeding to Liver-related death | 0.051 | 0.025 | 0.088 | Beta | 23.67 | 440.52 | [9] |
| Ascites with variceal bleeding to Encephalopathy | 0.185 | 0.061 | 0.339 | Beta | 20.19 | 88.95 | [9] |
| Ascites with variceal bleeding to HCC | 0.027 | 0.014 | 0.049 | Beta | 24.30 | 875.63 | [10] |
| Ascites with variceal bleeding to Liver-related death | 0.195 | 0.088 | 0.333 | Beta | 19.93 | 82.28 | [9] |
| Encephalopathy to HCC | 0.027 | 0.014 | 0.049 | Beta | 24.30 | 875.63 | [10] |
| Encephalopathy to Liver-related death | 0.320 | 0.157 | 0.527 | Beta | 16.68 | 35.45 | [9] |
| HCC to Liver-related death | 0.623 | 0.224 | 0.921 | Beta | 8.80 | 5.33 | [11] |
| Background mortality | China Statistical Yearbook 2021 | | | - | - | - | [1] |
| Abbreviations: PSA: probabilistic sensitivity analysis; F0: no fibrosis; F1: mild fibrosis; F2: moderate fibrosis; F3: advanced fibrosis; F4: compensated cirrhosis; HCC: hepatocellular carcinoma | | | | | | | |

| **Fig S1. Comparison of model predictions and GBD reported values.** |
| --- |
| **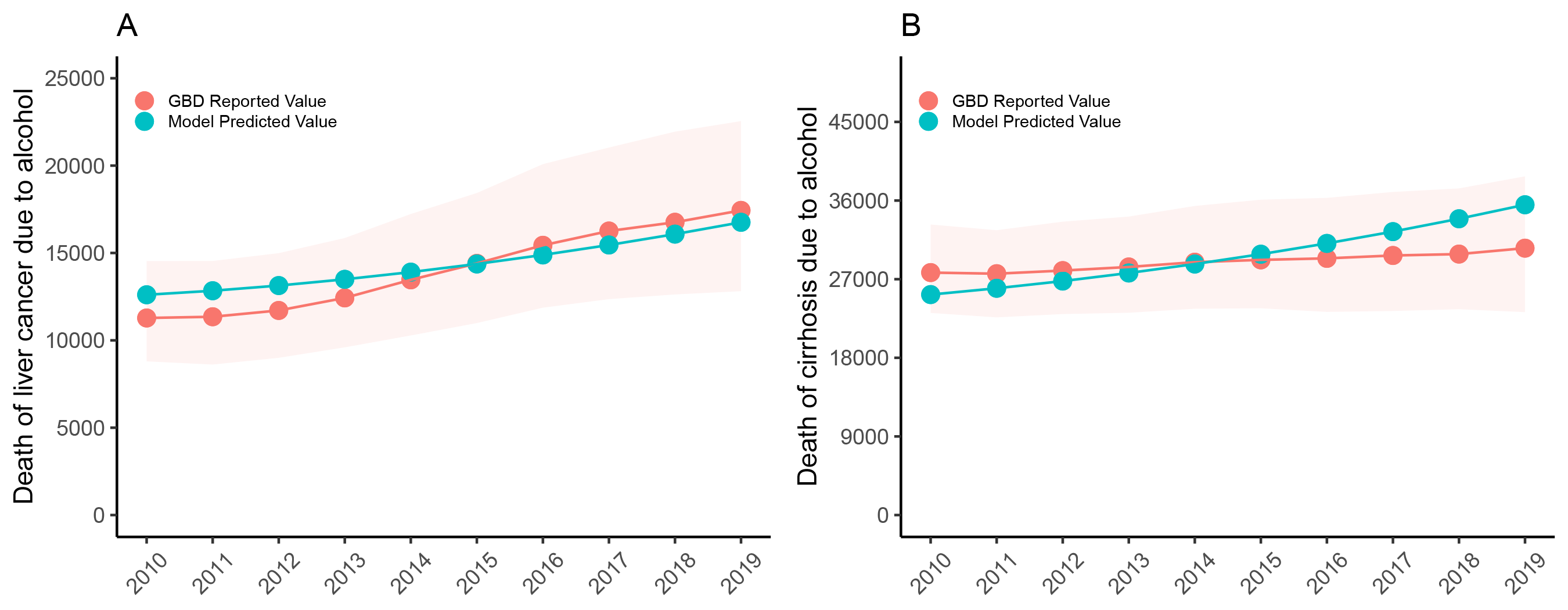** |
| (A) Comparison of model predictions with GBD reported deaths of liver cancer due to alcohol use; (B) Comparison of model predictions with GBD reported deaths of cirrhosis and other chronic liver diseases due to alcohol use. The shaded area represents the 95% uncertainty interval of the GBD reported value. It is worth noting that there is a discrepancy between the model predictions and GBD reported values, as the former focuses on alcohol-related cirrhosis that includes all outcomes for which alcohol can have an impact, whereas the GBD estimates of cases are only attributable to alcohol. |
|  |

| **Fig S2. The number and prevalence of excessive alcohol consumption for all projected scenarios** |
| --- |
| **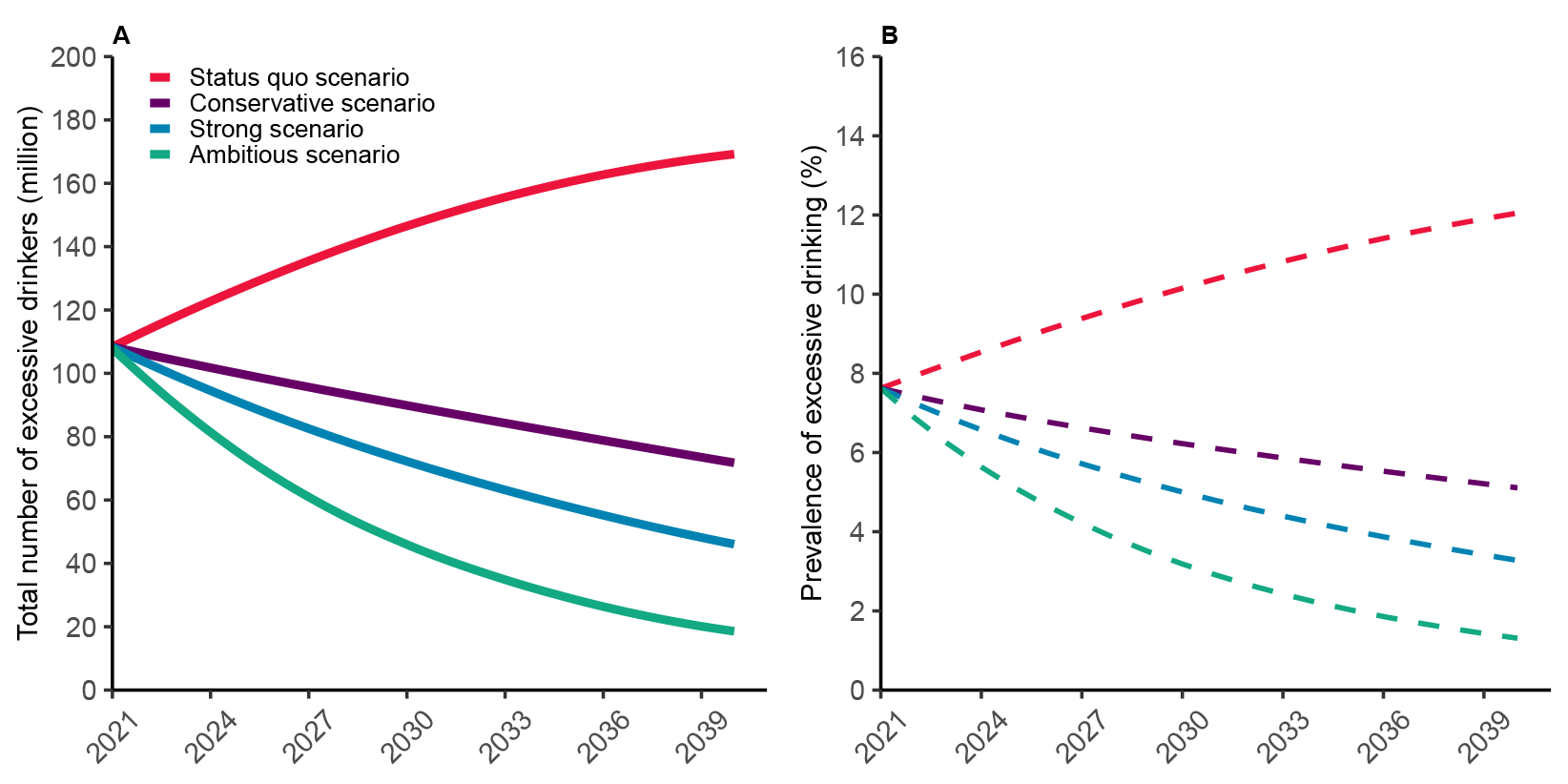** |
| (A) Annual number of excessive drinkers; (B) Prevalence of excessive drinking. |

# References

1. National Bureau of Statistics. China statistical year book 2021.

2. United Nations. World Population prospects 2022. https://population.un.org/wpp/Download/Standard/Interpolated/. Accessed 25 May 2023..

3. Asphaug L, Thiele M, Krag A, Melberg HO. Cost-Effectiveness of Noninvasive Screening for Alcohol-Related Liver Fibrosis. HEPATOLOGY. 2020 2020-06-01;71(6):2093-104.

4. Li YM, Fan JG. Guidelines of prevention and treatment for alcoholic liver disease (2018, China). J Dig Dis. 2019 2019-04-01;20(4):174-80.

5. Julien J, Ayer T, Bethea ED, Tapper EB, Chhatwal J. Projected prevalence and mortality associated with alcohol-related liver disease in the USA, 2019-40: a modelling study. Lancet Public Health. 2020 2020-06-01;5(6):e316-23.

6. Xu X, Zhao L, Fang H, Guo Q, Wang X, Yu W, et al. Status of alcohol drinking among population aged 15 and above in China in 2010-2012. Wei Sheng Yan Jiu. 2016 2016-07-01;45(4):534-67.

7. MA G, DU S, HAO L, LI Y, HU X, KONG L. THE PREVALENCE OF HEAVY DRINKING AMONG ADULTS IN CHINA. Acta Nutrimenta Sinica,Jun. 2009;31(03):213-7.

8. Poynard T, Mathurin P, Lai CL, Guyader D, Poupon R, Tainturier MH, et al. A comparison of fibrosis progression in chronic liver diseases. J HEPATOL. 2003 2003-03-01;38(3):257-65.

9. Jepsen P, Ott P, Andersen PK, Sorensen HT, Vilstrup H. Clinical course of alcoholic liver cirrhosis: a Danish population-based cohort study. HEPATOLOGY. 2010 2010-05-01;51(5):1675-82.

10. Kanwal F, Khaderi S, Singal AG, Marrero JA, Loo N, Asrani SK, et al. Risk factors for HCC in contemporary cohorts of patients with cirrhosis. HEPATOLOGY. 2022 2022-03-01.

11. Wang CY, Li S. Clinical characteristics and prognosis of 2887 patients with hepatocellular carcinoma: A single center 14 years experience from China. Medicine (Baltimore). 2019 2019-01-01;98(4):e14070.
